# Model-based analysis of competing-endogenous pathways (MACPath) in human cancers

**DOI:** 10.1371/journal.pcbi.1006074

**Published:** 2018-03-22

**Authors:** Hyun Jung Park, Soyeon Kim, Wei Li

**Affiliations:** 1 Department of Human Genetics, Graduate School of Public Health, University of Pittsburgh, Pittsburgh, Pennsylvania, United States of America; 2 Center for Precision Health, School of Biomedical Informatics, University of Texas Health Science Center, Houston, Texas, United States of America; 3 Division of Biostatistics, Dan L Duncan Cancer Center, Baylor College of Medicine, Houston, Texas, United States of America; 4 Department of Molecular and Cellular Biology, Baylor College of Medicine, Houston, Texas, United States of America; University of Calgary Cumming School of Medicine, CANADA

## Abstract

Competing endogenous RNA (ceRNA) has emerged as an important post-transcriptional mechanism that simultaneously alters expressions of thousands genes in cancers. However, only a few ceRNA genes have been studied for their functions to date. To understand the major biological functions of thousands ceRNA genes as a whole, we designed Model-based Analysis of Competing-endogenous Pathways (MACPath) to infer pathways co-regulated through ceRNA mechanism (cePathways). Our analysis on breast tumors suggested that NGF (nerve growth factor)-induced tumor cell proliferation might be associated with tumor-related growth factor pathways through ceRNA. MACPath also identified indirect cePathways, whose ceRNA relationship is mediated by mediating ceRNAs. Finally, MACPath identified mediating ceRNAs that connect the indirect cePathways based on efficient *integer linear programming* technique. Mediating ceRNAs are unexpectedly enriched in tumor suppressor genes, whose down-regulation is suspected to disrupt indirect cePathways, such as between DNA replication and WNT signaling pathways. Altogether, MACPath is the first computational method to comprehensively understand functions of thousands ceRNA genes, both direct and indirect, at the pathway level.

## Author summary

Competing endogenous RNAs (ceRNA) are RNAs known to co-regulate each other’s expression levels by competing for the binding of shared microRNAs. In human cancer, thousands genes are known to be in the ceRNA relationship. To understand the major biological functions of the ceRNA genes as a whole, we offer Model-based Analysis of Competing-endogenous Pathways (MACPath). Based on a network model representing ceRNA relationship changes in tumor, MACPath identifies biological pathways co-regulated through the ceRNA relationship (cePathways), since a biological pathway represents a group of genes coordinating to carry out the same biological function. In breast tumor data, MACPath shows that extensive ceRNA relationship changes in tumor are associated with previously under-characterized tumorigenic crosstalk e.g. between nerve growth factor and other tumor-related growth factor pathways. MACPath also identified indirect cePathways, whose ceRNA relationship is mediated by mediating ceRNAs. When usually thousands ceRNAs mediate indirect cePathways, MACPath employed efficient integer linear programming technique to characterize the mediation. In the breast tumor data, the characterization provides insights into how indirect cePathways and the mediating ceRNAs promote the tumorigenic process. To the best of our knowledge, MACPath is the first computational framework that can comprehensively identify cePathway relationship, both direct and indirect.

This is a *PLOS Computational Biology* Methods paper.

## Introduction

Competing endogenous RNAs (ceRNAs) are transcripts that share microRNA (miRNA) binding sites. Through competition for the binding of shared miRNAs, ceRNAs co-regulate each others’ expression levels[1] (ceRNA relationship). Although its scope is not fully understood[2], ceRNA has been shown to play a prominent role in regulating gene expression in diverse physiological[3, 4] and pathological conditions, especially for human cancers (reviewed in [5]). Tumor-associated ceRNA function has been identified for a number of individual genes. For example, as *CNOT6L* and *VAPA* have ceRNA relationship with *PTEN*, they regulate *PTEN* itself and phenocopy its tumor suppressive properties[6].

Recently, several studies reported widespread ceRNA relationship (ceRNA network) in tumors involving thousands ceRNA genes[7, 8]. To identify the tumor-associated function of thousands ceRNA genes as a whole rather than arbitrarily chosen individual genes, we developed Model-based Analysis of Competing-endogenous Pathways (MACPath). MACPath groups ceRNA relationships by competing-endogenous pathways (cePathway), i.e. pairs of biological pathways enriched with ceRNA relationships in-between. Since a biological pathway represents a group of genes coordinating to carry out the same biological function, a cePathway relationship may represent a co-regulation between biological functions (crosstalk) through enriched ceRNA relationships. Furthermore, MACPath also identifies dysregulated cePathway relationships between cancer and normal cells. For example, cePathway relationships gained in tumors might be oncogenic, while those lost in tumors might be tumor-suppressive. Finally MACPath can identify dysregulation in indirect cePathway relationship, i.e. co-regulation between pathways mediated by (direct) ceRNAs[9, 10]. When applied to TCGA breast cancer data, MACPath discovered thousands of lost and gained (direct and indirect) cePathway relationships, which collectively provide novel biological insights into ceRNA regulations during tumorigenesis.

## Results

### Direct cePathways dysregulated in tumors

**Fig 1:**
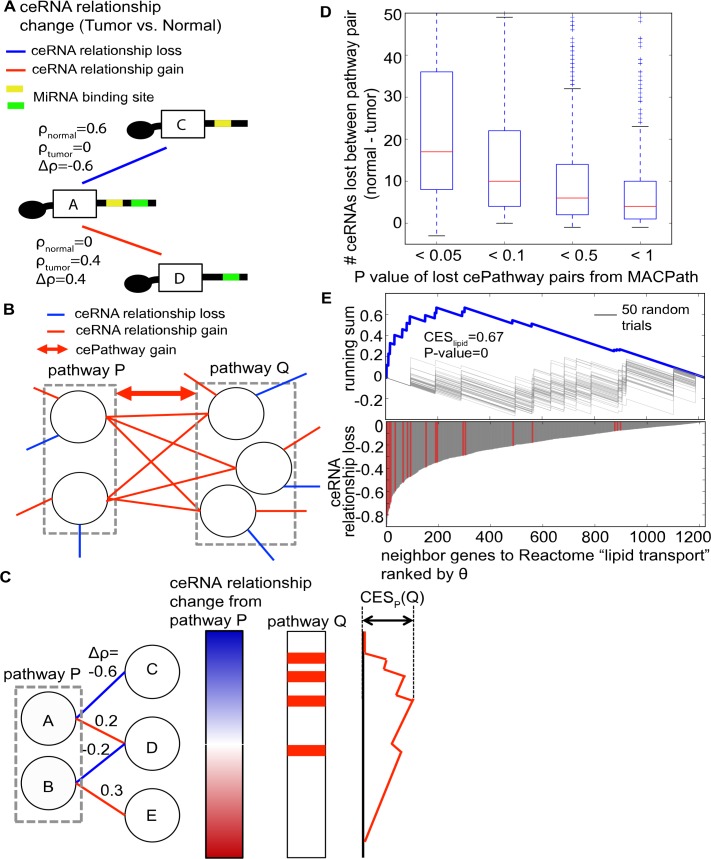
Identifying direct cePathway relationship dysregulations in tumors. **(a).** An illustration of ceRNA relationship change network. RNA A shares a significant number of miRNA binding sites with RNA C and D, where ρ_normal_ and ρ_tumor_ represent their co-expression in normal and tumor samples, respectively. Between normal and tumor samples, co-expression decrease in tumors (Δρ = ρ_tumor_-ρ_normal_<0) between A and C could represent their ceRNA relationship loss and co-expression increase (Δρ>0) between A and D represent their ceRNA relationship gain. **(b)**. An example of a cePathway relationship gain between pathway P and Q in the ceRNA relationship change network. CePathway relationship loss would be identified in the same fashion.**(c).** An illustration of MACPath to identify cePathway relationship dysregulation between pathway P and Q. Genes showing ceRNA relationship changes from pathway P are sorted by the magnitude of the changes (θ_P_ from **Def. 2**, blue for loss and red for gain in the left bar). On the genes ranked by θ_P_, a running sum is calculated for genes in pathway *Q* (in the right bar). The maximum variation of the running sum from 0 is the ceRNA relationship change enrichment score (CES_P_(Q)). **(d).** The number difference of ceRNAs between pathway pairs (normal vs. tumor) divided by P value MACPath estimated for the pairs. **(e).** Upper panel shows behavior of running sum between Reactome "lipid transport" pathway and PID "HIF1 TF" pathway. Gray lines represent running sum of 50 permutation trials. Lower panel shows θ of neighbor genes to Reactome "lipid transport" pathways ranked by their θ. Red lines indicate where genes in PID “HIF1 TF” are placed.

To identify direct cePathway relationships dysregulated in tumors, MACPath first builds a network model representing changes in ceRNA relationship between cancer and normal tissues (ceRNA relationship change network, **Def. 1**). Since co-expression between genes that share the same miRNA binding sites indicates the level of their ceRNA relationship[7, 10], their co-expression change (tumor vs. normal) can represent their ceRNA relationship change (**Fig 1A)**. In the network model, MACPath identifies dysregulated cePathway relationship between pathway P and Q (**Fig 1B**, for example) in three steps. First, MACPath calculates the ceRNA relationship changes between all genes within pathway P and their ceRNA genes. Second, MACPath estimates if pathway Q is over-represented in the ceRNA relationship changes (ceRNA relationship change enrichment score (CES_P_(Q)), **Fig 1C**). Third, MACPath estimates the statistical significance (empirical p-value) of the CES score by running a permutation test (**see** **Materials and Methods**). Pathways P and Q can gain cePathway relationship if they are significantly over-represented in the ceRNA relationship gains in tumors (**Fig 1B**, for example), or can lose cePathway relationship if over-represented in the ceRNA relationship losses.

In 97 breast tumor and their matched normal samples in TCGA, MACPath identified 1,019 lost and 1,698 gained cePathway relationships (FDR-corrected P value < 0.01 and CES > 0.6, **see** **Materials and Methods**) from all combinations (1330*1329/2 = 883,785) of 1,330 mSigDB canonical pathways[11] (**S1 Table**). To validate the use of MACPath, we identified 377,446 and 36,320 ceRNAs in the normal and tumor data based on co-expression (>0.6 in Pearson's correlation coefficient) and miRNA binding site share (< 0.05 in B-H corrected P-value)[7]. Since ceRNAs between pathways were suggested to indicate crosstalk between pathways[7], checking the ceRNA number difference from normal to tumor would be another way to represent cePathway dysregulation. In particular, pathway pairs losing ceRNAs in-between may represent lost cePathways, and those gaining ceRNAs represent gained cePathways. However, while MACPath generally identified pathway pairs losing many ceRNAs as lost cePathways, some lost cePathways do not lose many ceRNAs (43.3% of the lost cePathways lost less than 10 ceRNAs, **S1 Table, Fig 1D**). For example, Reactome "lipid transport" lost cePathway relationship with PID "HIF1 TF" pathway with a strong over-representation in the ceRNA relationship loss in tumor (P-value = 0, **Fig 1E**). However, they lost only 2 ceRNAs in tumor. Further, the ceRNA number difference cannot effectively identify gained cePathways, since 10-fold decrease of ceRNAs in tumor (377,446 vs. 36,320) makes it very difficult for pathway pairs to gain ceRNAs (only 8 of the 1,698 gained cePathways (0.4%) gain ceRNAs). Additionally, the ceRNA number difference cannot control for false positives, since it does not estimate significance. Altogether, MACPath identifies statistically significant and biologically reasonable cePathway dysregulation, which is impossible with a naive, yet the only other available method.

### Dysregulated cePathways may underlie NGF-induced breast tumor cell proliferation

**Fig 2:**
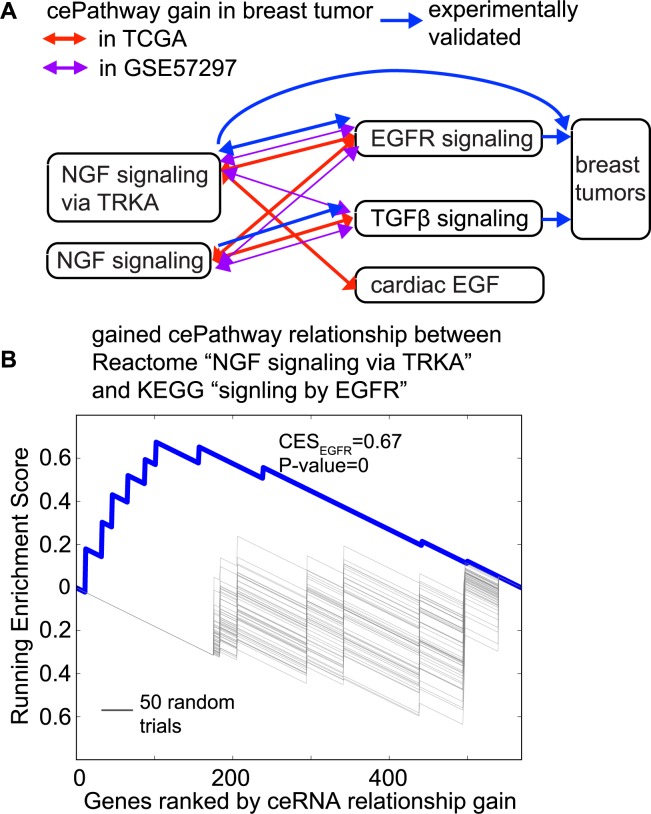
Dysregulated cePathways suggest NGF-induced cell proliferation. **(a).** Growth factor pathways that gained cePathway relationship in tumors with NGF signaling pathways in the TCGA data (red) and in GSE57297[19] (purple). **(b).** Behavior of running sum between Reactome “signaling by NGF” pathway and KEGG “TGFβ signaling” pathway. Gray lines represent running sum of 50 random trials (Actual p-values are estimated from 2,000 such random trials).

Many of the gained cePathway relationships involve nerve growth factor (NGF) signaling and tumor-related growth factor pathways, such as TGFβ[12] (transforming growth factor β) and EGFR[13] (epidermal growth factor receptor) (**Fig 2A**). An example is the gained cePathway relationship between REACTOME “NGF signaling via TRKA” and REACTOME “signaling by EGFR” (**Fig 2B**). NGF stimulates cell proliferation in tumor, but not in normal cells[14]. The mechanism may involve miRNAs, since NGF interacts with miRNAs for cell survival[15], which usually co-occurs with cell proliferation in cancer cells[16]. Therefore, we speculate that the mechanism underlying the NGF-induced cell proliferation might be due to the gained cePathway relationships between NGF and growth factor pathways. This is consistent with previous literature, in which crosstalk between NGF/TrkA and EGFR has been reported for cell activation[17], and NGF has been reported to up-regulate TGFβ in a cell differentiation experiment[18]. Furthermore, most of the cePathway gains in TCGA data are also found in an independent breast tumor data (GSE57297)[19] using MACPath with the same cutoff (**Fig 2A, see** **Materials and Methods**), suggesting the cePathway crosstalk between NGF and the growth factors is a common mechanism in breast cancers.

### Indirect cePathways dysregulated in tumors

**Fig 3:**
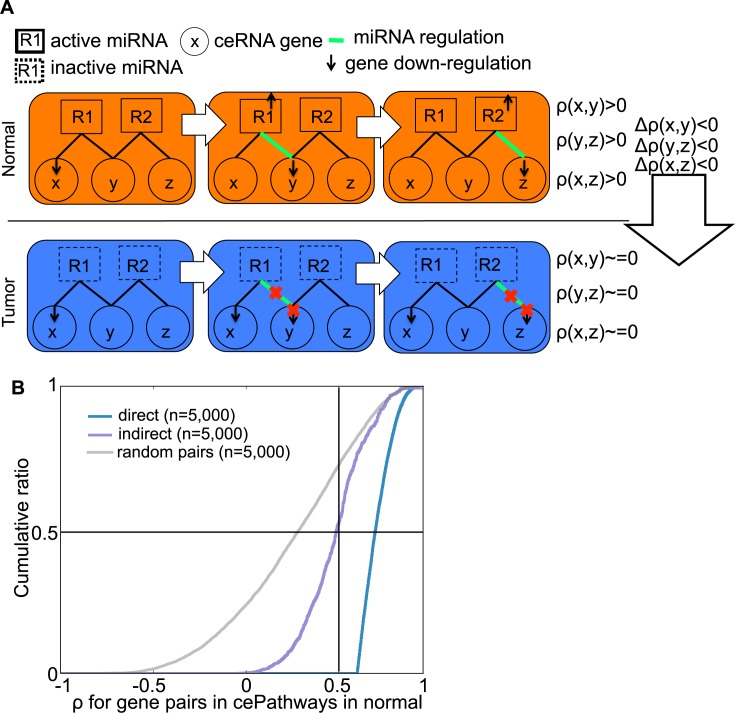
**Identifying gained/lost indirect cePathway relationships (a).** In normal when regulating miRNAs (R1 and R2) are active (in abundance relative to other competing molecules[21]), down-regulation of x would direct more copies of R1 to repress y. In turn, repressed y would direct R2 to repress z. Overall, ceRNA relationship on (*x*, *y*) and (*y*, *z*) would yield indirect ceRNA relationship on (*x*, *z*). In tumor when (*x*, *y*) lost ceRNA relationship possibly in association to inactivation of the regulating miRNAs, down-regulation of x would not repress y and z, representing the loss of not only direct ceRNA relationship in (*x*, *y*) and (*y*, *z*) but also indirect ceRNA relationship in (*x*, *z*). From normal and tumor condition, (*x*, *z*) would lose co-expression in tumors (Δρ<0) altogether with (*y*, *z*) and (*x*, *z*). **(b).** Accumulative distribution of co-expression (ρ) in direct and indirect ceRNA relationships (found in direct and indirect cePathway relationships) in normal samples, compared to the same number of random pairs (n = 5,000).

The ceRNA relationship change network that models direct ceRNA relationship (**Def. 1**) can further be used to model indirect ceRNA relationship. For genes x, y, and z, direct ceRNA relationship (x, y) can propagate to direct ceRNA relationship (y, z), making an indirect ceRNA relationship (x, z)[9, 10] (e.g. x, y, and z in normal condition in **Fig 3A**). Inspection on the same number (n = 5,000) of the ceRNA relationships showed that indirect ceRNA relationships are substantially co-expressed (ρ>0.5 for 53.8% of the relationships, **Fig 3B**), although less co-expressed than direct ceRNA relationships that are identified based on high co-expression. Additionally, indirect ceRNAs are more highly expressed than direct ceRNAs in the normal samples (P-value = 8.04e^-44^, **S2B Fig**). MACPath identifies indirect cePathway relationship based on indirect ceRNAs (x, z) that show the same direction of co-expression change as in (x, y) and (y, z) in the ceRNA network (**see** **Materials and Methods**). For TCGA breast cancer, MACPath identifies 509 lost and 84 gained indirect cePathway relationships (**S2 Table,** FDR-corrected P value <0.01 in ForwardStop[20]).

### Mediating ceRNAs drive indirect cePathway relationships

**Fig 4:**
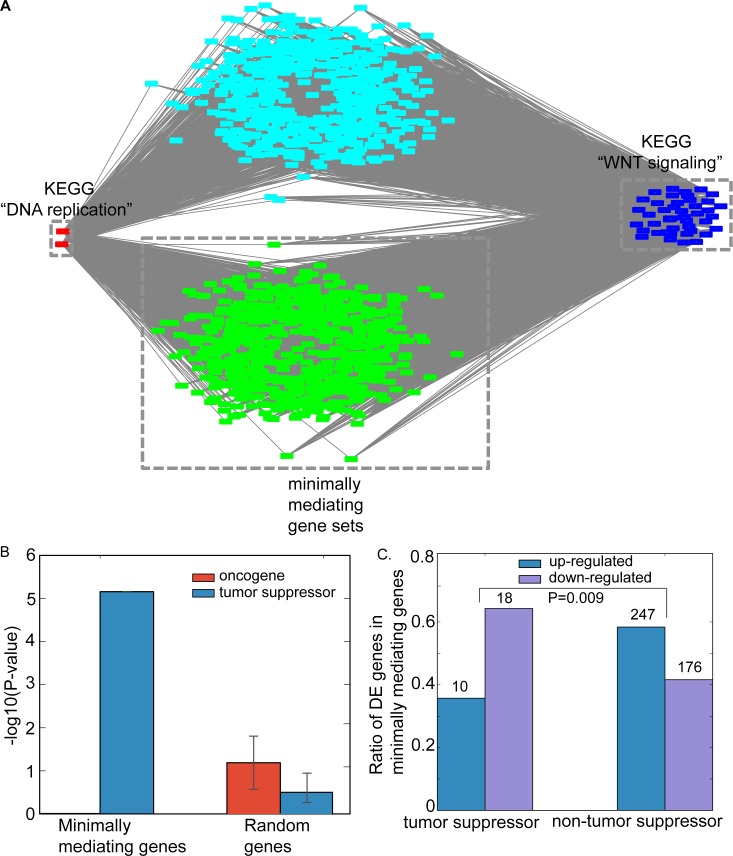
The lost indirect cePathway relationship between KEGG “DNA replication” and KEGG “WNT signaling”. **(a).** Red nodes are ceRNA genes of KEGG “DNA replication” and blue nodes are those of KEGG “WNT signaling”. 1,132 genes (green and light blue nodes) mediate the ceRNA loss between the pathways, where green nodes are 615 minimally mediating gene set. Visualization is taken from Cytoscape[27]. **(b).** Enrichment of the minimally mediating genes to oncogenes (red) and tumor suppressors (blue) compared to the same number of random genes. Error bar represents standard variation values from 100 random trials. **(c).** Ratio of differentially expressed tumor suppressors in the set of the mediating genes compared to other genes in the set. EdgeR estimates differential expression with FDR-corrected P value < 0.05.

Extensive ceRNA relationship in tumors[7, 8] implies that many genes would mediate indirect ceRNA relationships. Consistently, our network model identifies on average 840 mediating ceRNAs for each indirect cePathway relationship (**S3 Table**). For example, 1,132 genes are inferred to mediate the significant loss of indirect cePathway relationship between KEGG “DNA replication” and KEGG “WNT signaling” (P = 0.0005, **S2 Table**, **Fig 4A**). In normal cells, both WNT signaling and DNA replication were reported to crosstalk to the same genes, such as those in DNA damage pathways[22, 23], which would yield indirect crosstalk between DNA replication and WNT signaling. In addition, these two pathways were shown to suppress tumor in a miRNA dependent manner[24, 25], possibly through their indirect cePathway relationship.

One important question is to identify which of the 1,132 mediating ceRNAs would exert strong mediation effect for the loss of indirect cePathway. We address this problem by finding the minimum number of mediating genes covering all genes in the indirect cePathways. Finding minimally mediating genes is an NP-hard problem (**see** **Materials and Methods**), which requires trying all combinations of mediating genes. We formulated the problem such that it is amenable to efficient *integer linear programming* (ILP) solvers (**see** **Materials and Methods**) and found minimally mediating genes within a second for most indirect cePathway relationship dysregulation. In particular, MACPath employed GNU Linear Programming Kit (GLPK) solver, which returns a different set of minimally mediating genes for a different permutation of the same input values. Using the solver on multiple (10,000) permutations on the input values for the lost indirect cePathway relationship KEGG "DNA replication" and KEGG "WNT signaling", MACPath yielded 615 minimally mediating genes, which are surprisingly enriched for tumor suppressors, but not oncogenes (**Fig 4B**). Tumor suppressors that are also minimally mediating genes are more likely to be down-regulated than other non-tumor-suppressor minimally mediating genes (P-value = 0.009, **Fig 4C**). While tumor suppressor down-regulation is a hallmark of cancer[26], one of its tumorigenic effects may be to disrupt indirect cePathway relationships, for example between KEGG "DNA replication" and KEGG "WNT signaling".

### YAP, TAZ, and vitamin D receptor promote or inhibit lung adenocarcinoma possibly through ceRNA

To ascertain its general applicability, we ran MACPath on 58 TCGA lung adenocarcinoma (LUAD) and their matched normal samples, identifying direct cePathway dysregulation (13,247 gains and 7,121 losses in tumor) (**S3 Fig, S4 Table**). Further analyses on the dysregulation provide new insights into some tumor-associated crosstalks in lung adenocarcinoma (AC). Reactome “YAP1 and TAZ stimulated gene expression” that gained the most number (339) of cePathway relationships is an example. Overexpression of YAP and TAZ promotes growth signals in cancer types including lung AC [28]. Their expressions were found to be regulated by miRNAs in diverse cancer types [29], but their post-transcriptional tumorigenic mechanisms remain elusive. Since Reactome “YAP1 and TAZ stimulated gene expression” gains cePathway relationships with several tumor-associated growth factor pathways (e.g. EGFR, TGF*β* and IGF), YAP and TAZ would promote the growth factor pathways possibly through ceRNA relationships. Further, cePathway dysregulations suggest their potential roles as prognostic biomarkers. As an example, vitamin D receptor pathways gained many cePathway relationships (5^th^ and 11^th^ highest in the number of gains). Vitamin D receptor is known to regulate cell cycle [30], whose high expression correlating with longer survival through antiproliferative effects and cell cycle arrest [31]. Since its function as cell cycle regulator involve miRNAs in diverse types of cancers [30], vitamin D receptor pathways gaining cePathway relationships to 23 cell cycle checkpoint pathways may enhance the antiproliferative and cell cycle arrest mechanisms at the ceRNA level.

## Discussion

CeRNA plays an important role in the pathogenesis of cancers by forming extensive ceRNA network (ceRNET)[32] involving thousands ceRNA genes[21, 33]. For the ceRNETs to produce appropriate tumor-associated responses, the ceRNA genes are expected to coordinate to function in the level of biological pathways. Therefore, identifying ceRNA relationship between biological pathways (cePathway) would be a useful tool to understand tumor-associated ceRNA functions as a whole. For this purpose, we developed a computational method MACPath that identifies pathway pairs enriched in a differential network that encodes the ceRNA relationship changes (gain/loss) in tumor (cePathway relationship gain/loss).

A few computational methods have been proposed to identify pathway pairs enriched in a differential network[34, 35], which can be adapted to identify direct cePathway relationship dysregulation. However, they are not directly applicable for indirect cePathway relationships, since they do not consider indirect relationships. To the best of our knowledge, MACPath is the first computational method that can comprehensively identify both direct and indirect cePathway relationship dysregulation in a unified computational platform.

## Materials and methods

### MiRNA binding site database

Predicted miRNA binding sites were obtained from TargetScanHuman version 6.2[36]. For accurate analyses, those with preferentially conserved targeting score (Pct) more than 0 were used. Experimentally validated miRNA binding sites were obtained from TarBase version 5.0[37], miRecords version 4[38], and miRTarBase version 4.5[39]. The binding sites found in indirect studies such as microarray experiments and high-throughput proteomics measurements were filtered out[40]. Another source is microRNA target atlas composed of public AGO-CLIP data[41]. As suggested, we employed only significant binding sites (q-value < 0.05). The predicted and validated binding site information was combined for use[40]. Note that all miRNAs are converted to miRNA family ID[36], and representative miRNA names for the families (appeared first in the ID) are presented.

### TCGA breast tumor RNA-seq data

Quantified gene expression files (RNASeqV1) for 97 primary breast tumors (TCGA sample code 01) and their matching normal samples (TCGA sample code 11) were downloaded from TCGA Data Portal[42]. Tumor samples are composed of various subtypes. 10,868 expressed RefSeq genes (FPKM ≥ 1 in > 80% of all samples) were selected for downstream analyses. To better quantify gene expression in the presence of 3′ UTR-shortening, we only counted reads in the coding regions, approximated by taking maximum CDS region for each exon across different CDS annotations of multiple transcripts. Exon and CDS annotation for TCGA data were downloaded from Sage Bionetworks’ Synapse database system.

### CeRNA relationship change network

Given gene expression levels in normal and tumor samples, MACPath first builds ceRNA relationship change network in the following.

**Definition 1**. CeRNA relationship change network *G* is an undirected graph *(V*, *E)*, where *V* is a set of genes and *E* ⊂ *V* × *V* represents gene pairs with significant miRNA binding site overlap (**S1A Fig**).

Since, for a gene pair with significant miRNA binding site overlap (*x*,*y*) ∈ *E*, co-expression increase/decrease in tumors would represent ceRNA relationship gain (Δ*ρ*_*tumor–normal*_(*x*,*y*) > 0) or loss (Δ*ρ*_*tumor–normal*_(*x*,*y*) < 0), *G* would encode ceRNA relationship change by holding the co-expression change value for (*x*,*y*) ∈ *E*. In building *G* for the breast tumor data (**S1B and S1C Fig**), we used only expressed mRNAs (FPKM>1) and miRNAs with moderate expression (FPKM between 0.01 and 100), since these are known to facilitate active ceRNA relationship[10]. After removing genes with less than 6 such miRNA binding sites, gene pairs with significant miRNA binding site overlap (< 0.05 in B-H corrected p-value) were selected, where significance is estimated from hypergeometric test.

### Identifying direct cePathway relationships gained/lost in tumors

Given the ceRNA relationship change network (**Def. 1**), MACPath assesses if pathway *P* and *Q* would gain or lose the cePathway relationship in tumors in the three steps.

#### Step 1. Estimating ceRNA changes

ceRNA relationship change between *P* and its neighbor gene *y* is measured.

**Definition 2.** θ_P_(y) = ∑_x∈P_ Δρ(x,y), if (x,y) ∈ E, where Δρ(x,y) = ρ_tumor_(x,y) − ρ_normal_(x,y).

θ_P_(y) represents gain or loss of co-expression (and thus ceRNA relationship) in tumors between y and *P*; negative values represent loss and positive values represent gain. θ_P_(*y*) is calculated for all genes connected to *P* in *G* and they are ranked by θ_P_ (**Fig 1C**).

#### Step 2. Calculating ceRNA relationship enrichment score

If pathway *Q* is not enriched in the neighbor genes, MACPath calls that they are not in a cePathway relationship. If pathway *Q* is enriched, we walk down the ranked list produced in Step 1, as a running sum is increased when we encounter a gene in *Q* and decreased when otherwise (**Fig 1C**). From the running sum, ceRNA relationship enrichment score (*CES*_*P*_*(Q)*) is the maximum deviation from zero. To avoid yielding high scores for *Q* that is clustered near the middle of the ranked list, the amount of increase is set proportional to θ_P_. However, the use of weighted increase (θ_P_) can cause an asymmetric distribution of CES scores, complicating the interpretation of the scores[11]. Therefore, we followed Subramanian *et al*.[11] and considered genes with the negative and positive θ_P_ values separately for lost and gained cePathway relationships, respectively.

#### Step 3. Estimating significance of CES

From 2,000 permutations on the gene labels in the ranked list, p-value is the fraction of the permutations where the calculated *CES*_*p*_ values are greater than or equal to the observed *CES*_*P*_.

For the TCGA breast tumor data, we selected significantly lost or gained cePathway relationships based on the CES values corresponding to top 5% of the empirical distribution (>0.6) and the multiple-testing corrected significance (B-H P value < 0.01).

### GEO breast tumor microarray data

To validate our findings from the TCGA breast tumor data using an independent data set, we downloaded gene expression profiles on 32 human breast tissues of multiple cancer subtypes (including 19 Luminal A, 3 Luminal B, 3 Triple Negative) and 7 (non-matched) normal controls in microarray data (GSE57297) [19]. Although TCGA RNA-Seq data contain matched normal samples, this data do not. We used the normalized signal intensity of genes provided in the series matrix file.

### Identifying indirect cePathway relationships gained/lost in tumors

To identify indirect cePathway relationship dysregulation in tumors, we define *κ*_*P*_(*z*), indirect ceRNA relationship change between pathway P and its indirect ceRNA partner *z* through mediating genes in the ceRNA relationship change network (**Def. 1**).

**Definition 3**. *κ*_*P*_(*z*) = ∑_*x*∈*P*_Δρ(x,z), if

i) (x,y) ∈ *E* and (y,z) ∈ *E* for some *y*,
ii) Δρ(x,y), Δρ(y,z), and Δρ(x,z) are in the same direction.

Since indirect ceRNA change between *x* and *z* would be propagated from ceRNA (*x*, *y*) and (*y*,*z*), the second condition filters out invalid indirect ceRNA changes. On the estimated indirect ceRNA changes using *κ*_*P*_(*z*), MACPath identifies indirect cePathway dysregulations by following **Step2** and **3** defined for direct cePathway relationship dysregulation.

### Controlling false discovery rate in an adaptive manner

Between direct and indirect cePathways, it would be more difficult to identify true signals for indirect cePathways, because it involves another dimension of data, mediating RNAs. To identify true signals for indirect cePathways in a more sophisticated way, we tested for FDR control in an adaptive way[43]. Among adaptive methods showing higher statistical power, we selected ForwardStop[20] with *α* = 0.01 to be stringent.

### Pathway annotation in the analyses

We annotate pathways based on keyword search in the following way: A(_B) pathways are identified with keyword “A” (and “B”) in the corresponding table. For example, cancer pathways are with keyword “CANCER”. After collecting pathways with the keywords, manual inspection was conducted to filter out irrelevant pathways. Manual inspection indicated that the keyword-based identification mostly identified only relevant pathways.

### Minimum number of mediating genes for indirect cePathway relationship dysregulation

Assuming that all mediating genes cover genes both in *P* and *Q*, this problem is identical to Minimum Set Cover Problem (MSCP)[44]. For multiple sets covering items in a universe, MSCP finds the minimum number of sets that covers the whole universe. With each mediating gene taken as a set covering genes in *P* and *Q*, seeking the minimum number of sets covering *P* and *Q* is identical to MSCP. Hence, this problem is also NP-hard. We formulate the problem into *integer linear programming*, in which the objective function and the constraints are given as follows.

We define a binary variable as follows:

*B*_*pm*_, ∀*p* ∈ (*P* ∪ *Q*), ∀*m* ∈ *V* − (*P* ∪ *Q*). *B*_*pm*_ will take value 1 if (*p*,*m*) ∈ *E*. *B*_*pm*_ = 1 represents the case where gene *p* ∈ (*P* ∪ *Q*) is connected to *m*.

Then, the ILP program is:

Minimize |M|
Subject to *B*_*pm*_ = 1, ∀*p* ∈ (*P* ∪ *Q*), *m* ∈ *M*.

To solve based on the ILP program, we used GNU Linear Programming Kit (GLPK) solver (version 5.1.3). Although the GLPK solver does not return a comprehensive set of solutions, permutation on the input values returns multiple solutions of the same size. Based on the multiple solutions, we conducted functional analysis (**Fig 3B and 3C**).

### Tumor suppressors and oncogenes

The tumor suppressors and oncogenes used in this study were defined by the TUSON algorithm from genome sequencing of >8,200 tumor-normal pairs[45], namely residue-specific activating mutations for oncogenes and discrete inactivating mutations for tumor suppressors. TUSON is a computational method that analyzes patterns of mutation in tumors and predicts the likelihood that any individual gene functions as a tumor suppressor or oncogene. We ranked genes by their TUSON prediction P values from the most to the least significant and used the top 500 genes (P-value < 0.01) as the reference tumor suppressors or oncogenes. After removing 30 genes in common, 470 tumor suppressors and oncogenes were used for the enrichment analysis. Please note that there were very few breast tumor-specific tumor suppressors and oncogenes (36 and 3 with breast_q-value ≤ 0.5, respectively) and 90% of them were found in the top 500 pan-cancer predictions.

## Supporting information

S1 Fig CeRNA relationship change network for the TCGA breast tumor data. **(a).** Illustration of the pipeline to build the ceRNA relationship change network (**Def. 1**) for the TCGA breast tumor data. **(b).** Connectivity (number of neighbor genes) of genes in the network against the number of miRNA binding sites residing in their 3′UTR. **(c).** Distribution of Δ*ρ*_*tumor–normal*_ values in the network. 63.8% of edges lose co-expression (Δρ < 0) in tumors, marked by gray vertical line. Red line indicates Δρ value for the case of *r* = 0.01.
(TIF)

S2 Fig Indirect cePathway relationships. **(a).** An illustration of invalid indirect ceRNA change. In tumor samples (column), gene x, y, z are expressed (white for lowly expressed and black for highly expressed). If they were not correlated at all in the matched normal samples, co-expression change (tumor vs. normal) of (x,y) and of (y,z) would be positive with their correlation gain in the half of the tumor samples. However, co-expression of (x,z) would not be gained in tumor (thus, not functional), because (x,y) and (y,z) were correlated in the other half of the tumor samples (red rectangles). **(b).** Average expression levels of genes across normal samples (y-axis) belonging to direct ceRNAs and equally correlated (ρ > 0.6) indirect ceRNAs (t-statistic -14.1 and P-value 8.04e^-44^).
(TIF)

S3 Fig Number of cePathway partners gained or lost in TCGA lung adenocarcinoma tumor samples vs. matched normal. (TIF)

S1 Table Information of direct cePathways gained or lost in the TCGA breast cancer samples (vs. matched normal samples). (XLSX)

S2 Table Information of indirect cePathways gained or lost in the TCGA breast cancer cells (vs. matched normal cells). (XLSX)

S3 Table 615 minimally mediating ceRNAs and 517 other mediating ceRNAs connecting the lost indirect cePathway relationship KEGG "DNA replication" and KEGG "WNT signaling". (XLSX)

S4 Table Information of direct cePathways gained or lost in the TCGA lung adenocarcinoma samples (vs. matched normal samples). (XLSX)
