# Supplementary figures and images for: Model-based analysis of competing-endogenous pathways (MACPath) in human cancers
Source: PLoS Comput Biol. 2018 Mar 22;14(3):e1006074. doi: 10.1371/journal.pcbi.1006074 (PMC5882149; doi:10.1371/journal.pcbi.1006074)

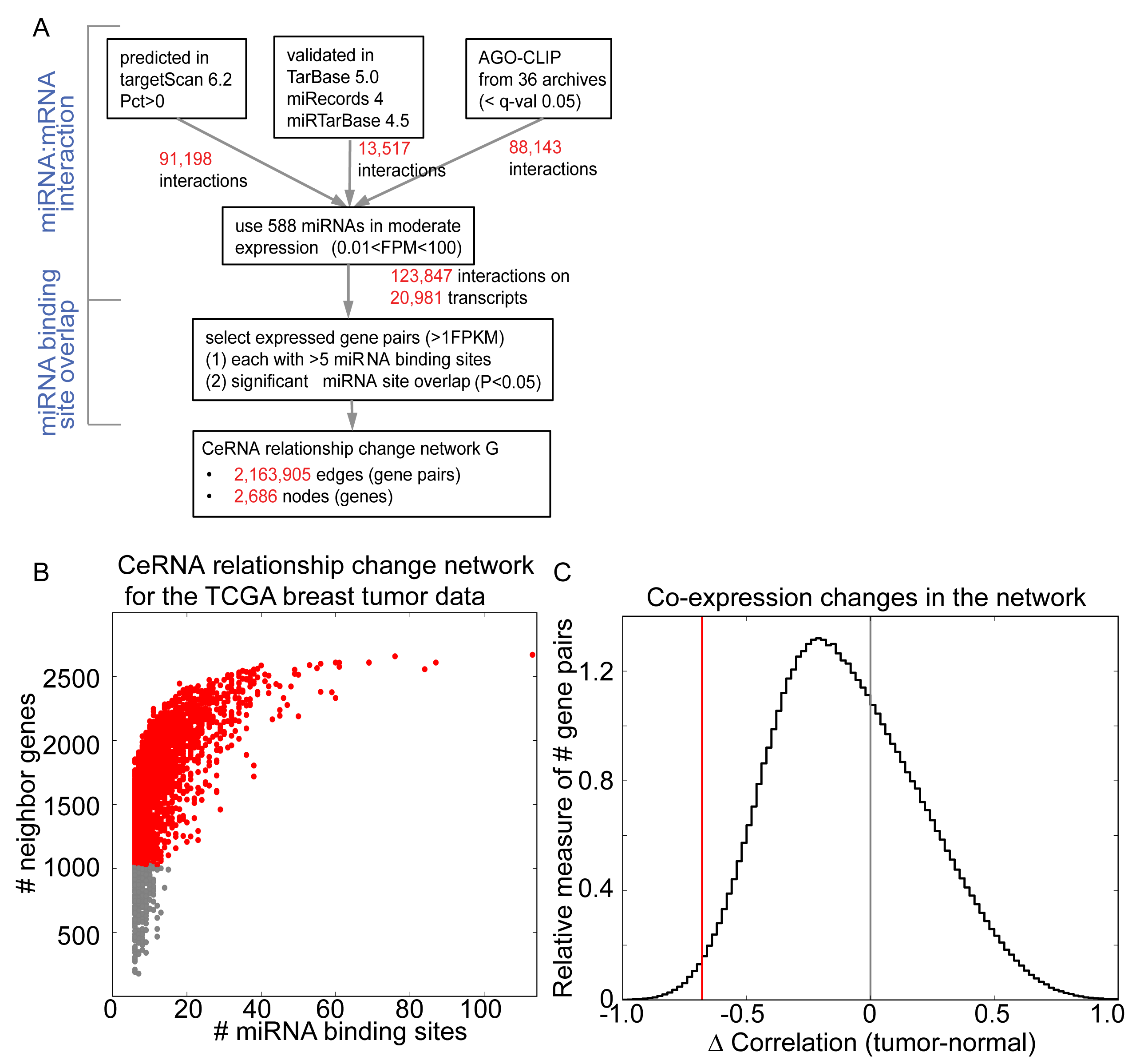

Supplement: S1 Fig — (a). Illustration of the pipeline to build the ceRNA relationship change network (Def. 1) for the TCGA breast tumor data. (b). Connectivity (number of neighbor genes) of genes in the network against the number of miRNA binding sites residing in their 3′UTR. (c). Distribution of Δρtumor–normal values in the network. 63.8% of edges lose co-expression (Δρ < 0) in tumors, marked by gray vertical line. Red line indicates Δρ value for the case of r = 0.01. (TIF) [file pcbi.1006074.s001.tif]

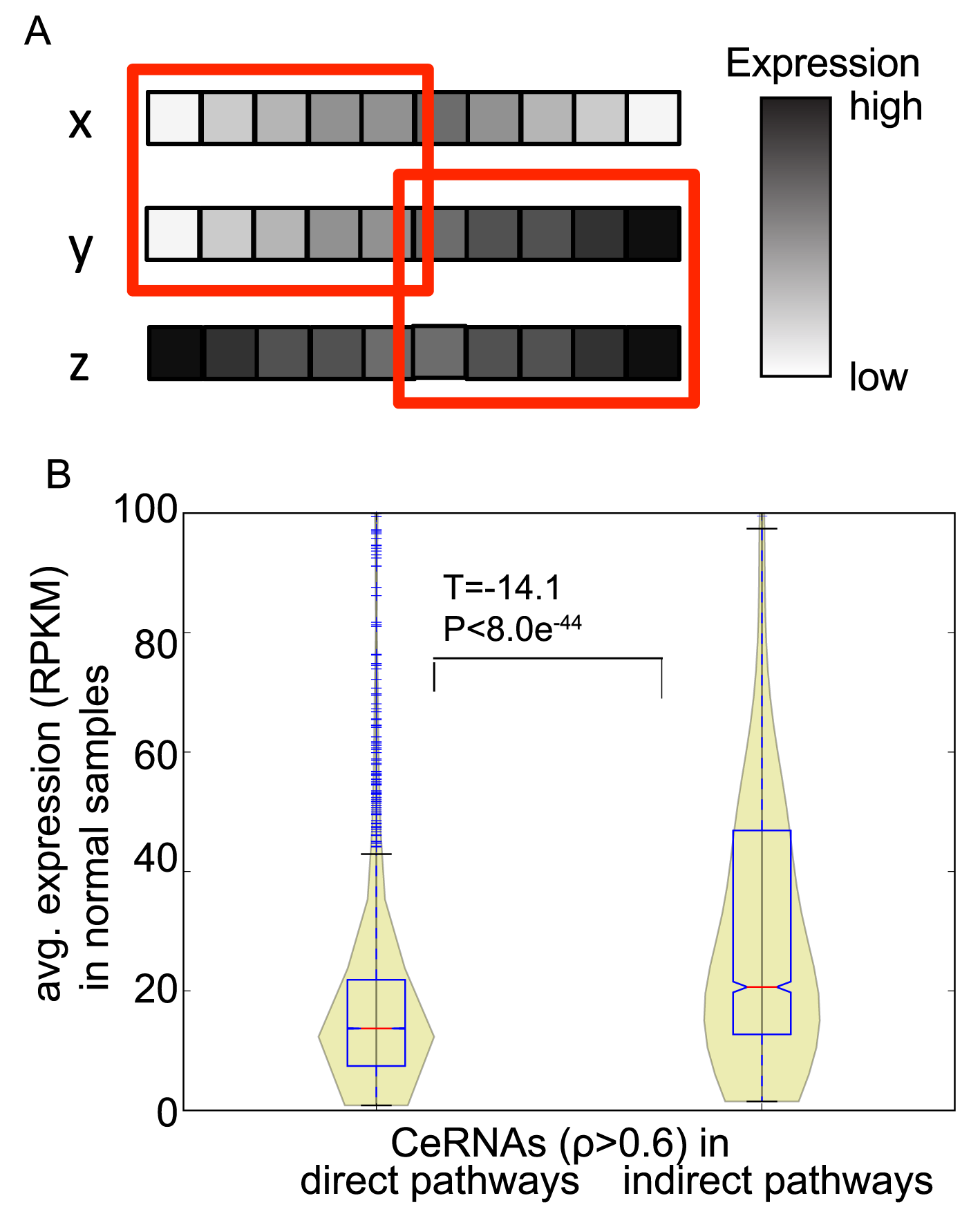

Supplement: S2 Fig — (a). An illustration of invalid indirect ceRNA change. In tumor samples (column), gene x, y, z are expressed (white for lowly expressed and black for highly expressed). If they were not correlated at all in the matched normal samples, co-expression change (tumor vs. normal) of (x,y) and of (y,z) would be positive with their correlation gain in the half of the tumor samples. However, co-expression of (x,z) would not be gained in tumor (thus, not functional), because (x,y) and (y,z) were correlated in the other half of the tumor samples (red rectangles). (b). Average expression levels of genes across normal samples (y-axis) belonging to direct ceRNAs and equally correlated (ρ > 0.6) indirect ceRNAs (t-statistic -14.1 and P-value 8.04e-44). (TIF) [file pcbi.1006074.s002.tif]

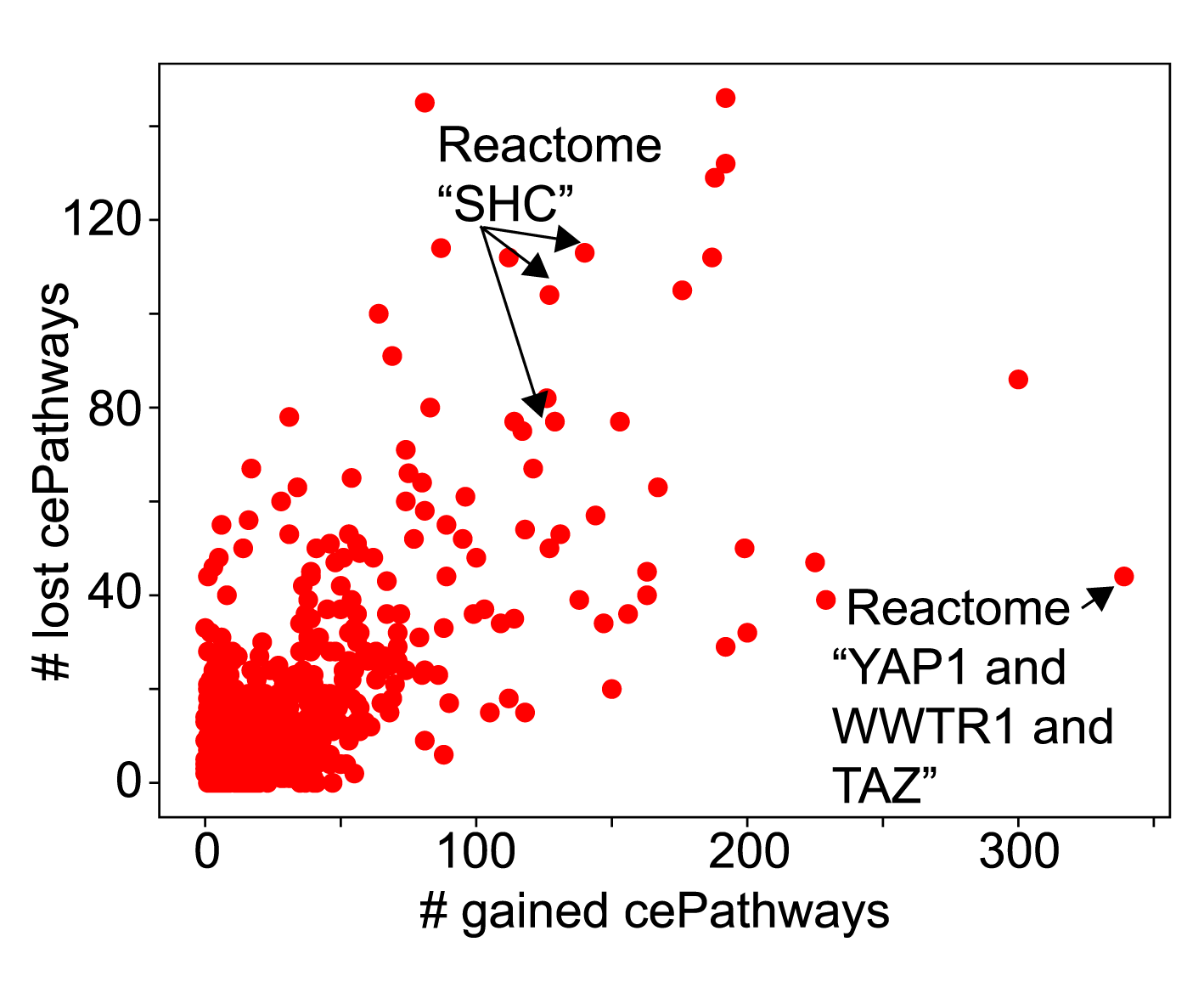

Supplement: S3 Fig — (TIF) [file pcbi.1006074.s003.tif]
